# MEK/ERK Dependent Activation of STAT1 Mediates Dasatinib-Induced Differentiation of Acute Myeloid Leukemia

**DOI:** 10.1371/journal.pone.0066915

**Published:** 2013-06-25

**Authors:** Yanfen Fang, Like Zhong, Meihua Lin, Xinglu Zhou, Hui Jing, Meidan Ying, Peihua Luo, Bo Yang, Qiaojun He

**Affiliations:** 1 Institute of Pharmacology and Toxicology, School of Pharmaceutical Sciences, Zhejiang University, Hangzhou, Zhejiang, China; 2 Department of Pathology, University of Michigan Medical School, Ann Arbor, Michigan, United States of America; Emory University, United States of America

## Abstract

Dasatinib (BMS-354825) is a FDA-approved multitargeted kinase inhibitor of BCR/ABL and Src kinases. It is now used in the treatment of chronic myelogenous leukemia (CML) with resistance or intolerance to prior therapies, including imatinib. Here we report a novel effect of dasatinib on inducing the differentiation of acute myeloid leukemia (AML) cells through MEK/ERK-dependent activation of signal transducer and activator of transcription 1 (STAT1). We found that dasatinib could induce the differentiation of AML cells as demonstrated by the expression of differentiation marker CD11b, G0/G1 phase arrest and decreased ratio of nucleus to cytoplasm. Of note, dasatinib induced robust phosphorylation of STAT1 both at Tyr701 and Ser727 as well as the redistribution of STAT1 from the cytoplasm to the nucleus, thus leading to the transcription of STAT1-targeted genes. Knocking down STAT1 expression by shRNA significantly attenuated dasatinib-induced differentiation, indicating an important role of STAT1 in myeloid maturation. We further found that dasatinib-induced activation of STAT1 was regulated by the MEK/ERK kinases. The phosporylation of MEK and ERK occurred rapidly upon dasatinib treatment and increased progressively as differentiation was induced. MEK inhibitors PD98059 and U0216 not only inhibited the phosphorylation of STAT1, but also abrogated dasatinib-induced myeloid differentiation, suggesting that MEK/ERK dependent phosphorylation of STAT1 might be indispensable for the differentiating effect of dasatinib in AML cells. Taken together, our study suggests that STAT1 is an important mediator in dasatinib-induced differentiation of AML cells, whose activation requires the activation of MEK/ERK cascades.

## Introduction

Acute myeloid leukemia (AML), characterized by a differentiation blockage and accumulation of immature myeloid cells, has been recognized as a heterogeneous disorder in clinic [1], [2]. The concept of differentiation therapy has been considered as a promising approach for the treatment of AML since 1970s [3]. In 1980s, the successful use of all-trans-retinoic acid (ATRA) in acute promyelocytic leukemia (APL) that elicited complete remission (CR) of more than 90% of APL patients has brought differentiation therapy from theoretical exploration to clinical application [4], [5]. Although great breakthrough in clinical oncology has been achieved by differentiation therapy with ATRA, ATRA-based therapy showed poor results in non-APL AMLs, thus tremendous efforts have been conducted to explore novel molecular targets as well as identify efficient differentiating compounds in AML treatment.

The off-target effects of tyrosine kinase inhibitors (TKIs) on inducing AML differentiation have been a topic of considerable interest in the last few years. Imatinib, the first BCR/ABL inhibitor, has been revealed to exert an unexpected effect on potentiating ATRA-induced AML differentiation [6]. The EGFR inhibitor gefitinib has also been demonstrated to enhance ATRA-induced differentiation of leukemic cells [7] and to trigger the differentiation of AML cell lines (HL60, kasumi-1 and U937) that lack the expression of EGFR [8] when used alone. Dasatinib, which targets a variety of tyrosine kinases, most notably the BCR/ABL fusion protein and the Src Family Kinases (SFKs), is used for the treatment of BCR/ABL+ CML and acute lymphocytic leukemia with imatinib resistance or intolerance to previous therapy [9], [10], [11], [12]. In vitro studies have shown that dasatinib significantly inhibited the growth of a variety of AML cell lines and primary blasts when treated alone or in combination with cytotoxic or molecular-targeted agents [13], [14]. Of note, dasatinib was also reported to promote ATRA-induced differentiation of AML cell lines and restore differentiating response of non-APL primary AML cells to ATRA, while dasatinib alone could not promote leukemic cell differentiation [15], [16]. In contrast, Lainey et al.’s study demonstrated that dasatinib by itself could overcome the AML-typical differentiation blockage as evidenced by dasatinib-induced differentiation of MOLM13 and HL60 cells [17]. This effect was further confirmed by a clinical study which provided proof of prolonged differentiation of myeloid blasts bearing the t(8;21) to mature after dasatinib treatment [18]. Inhibition of c-Kit and induction of CAAT-enhancer binding protein-α (C/EBPα) was demonstrated to contribute to this remarkable response-induced by dasatinib [18]. However, the mechanisms by which dasatinib trigger AML differentiation remains largely unknown.

In the present study, we first reported that dasatinib-induced differentiation of AML cells was accompanied with the activation of the signal transducer and activator of transcription 1 (STAT1), which was evidenced by the augmented phosphorylation of STAT1, the redistribution of STAT1 from cytoplasm to nucleus and the enhanced transcription of STAT1 direct target genes. We then showed that STAT1 knockdown significantly decreased the differentiating effect of dasatinib, indicating a potential role of STAT1 in dasatinib’s off-target effects. We further found that dasatinib-induced STAT1 activation was MEK/ERK cascade dependent since MEK inhibitors not only inhibited dasatinib-triggered phosphorylation of STAT1, but also abrogated dasatinib-induced differentiation of AML cells. In summary, our results suggested a novel mechanism associated with the differentiation-induction effect of dasatinib on leukemic cells, which may provide theoretical support for the anticancer approach of dasatinib in AML.

## Materials and Methods

### Cell Culture and Chemical

Human acute myeloid leukemia HL60 and NB4 cells were cultured in RPMI 1640 medium plus 10% heat-inactivated fetal bovine serum (FBS). Human embryonic kidney 293T cells were cultured in Dulbecco’s modified Eagle medium plus 10% FBS. All cell lines were purchased from Shanghai Institute of Biochemistry and Cell Biology (Shanghai, China), and grown in the medium supplemented with 100 units/mL penicillin and 100 µg/mL streptomycin at 37°C with 5% CO_2_. Dasatinib (purity: 99.1%) was a generous gift from Shanghai Institute of Materia Medica, Chinese Academy of Sciences (Shanghai, China). Nitroblue tetrazolium (NBT), PD98059, U0126, and AG490 were purchased from Calbiochem (San Diego, CA). All compounds were dissolved in DMSO and stocked at −20°C. In all experiments, the final DMSO solvent concentration was ≤0.2% (v/v).

### Cell Proliferation, Cell Cycle and Apoptosis Analysis

Cell proliferation and viability were assessed by manual counting using a standard hemocytometer following with trypan blue staining. Cell cycle distribution was determined by flow cytometry measurement of DNA content after cells were incubated with RNase A (10 µg/ml) and propidium iodide (50 µg/ml). The cellular DNA content was analyzed on a FACSCalibur flow cytometer using the CellQuest Pro software (BD Biosciences, San Jose,CA). The percentage of each population was measured using ModFIT software (BD Biosciences). At least 20 000 cells were analyzed for each data point. Apoptosis was detected by FITC Annexin V Apoptosis Detection Kit I according to the manufacturer’s instructions (BD Biosciences) to determine the phosphatidyl serine exposure. Apoptotic cells were quantified by FACSCalibur flow cytometer using the CellQuest Pro software. At least 10 000 cells were analyzed for each data point.

### Assessment of Cell Differentiation

Fluorochrome conjugate of monoclonal antibody CD11b-PE was used to detect CD11b expression following the protocol from the manufacturer (BD Biosciences). The stained cells were run on a FACSCalibur flow cytometer and quanititated using CellQuest Pro software. At least 10 000 cells were analyzed for each data point. Cells stained with mouse IgG-PE served as negative controls. For NBT reduction and morphology analysis, cytospin preparations were made using a Shandon cytospin 4 cytocentrifuge. NBT reduction was performed to examine for cells containing the precipitated formazan particles as described [19]. At least 200 cells were assessed for each experiment. Cell morphology was evaluated by Wright–Giemsa staining and examined under a Leica microscope and captured with a Leica DFC300 FX charge-coupled device camera.

### Immunofluorescence

HL60 cells treated with dasatinib or vehicle were collected and cytospined onto slides, fixed in 4% paraformaldehyde at room temperature. Cells were then permeabilized with 0.1% triton X-100 for 10 min, washed with PBS, and blocked with 10% FBS for 10 min. After that, cells were stained with the indicated primary antibodies-STAT1 (Santa Cruze, CA) overnight at 4°C, followed by Alexa Fluor 488-conjugated second antibody (Life Technologies Corp, Eugene, OR) and 4′, 6-diamidino-2-phenylindole (DAPI) for 1 h at room temperature. Each incubation step was followed by three washes with PBS. Images of stained cells were obtained with a Leica fluorescence microscope and a Leica DFC300 FX charge-coupled device camera [20].

### Real-time Quantitative PCR Assay

Total RNA was isolated from HL60 cells treated with dasatinib or vehicle, using the Trizol reagent (Bio Basic Inc., Markham, Ontario, Canada). RNA was transcribed into cDNA using random hexamer primers and RevertAidTM M-MuLV Reverse Transcriptase (Fermentas International Inc., Burlington, Ontario, Canada). Equilibrated amounts of cDNA were taken for transcript PCR amplification, which was performed using SYBR Premix Ex Taq (Takara Biotechnology, Dalian, China). The housekeeping gene GAPDH was used as an internal standard. Primer sequences used for the PCR were as follows: RIG-G: Forward 5′-AACTACGCCTGGGTCTACTATCACT-3′, Reverse 5′-ACACCTTCGCCCTTTCATTTC-3′ [21]; CXCL-10: Forward 5′-GAATCGAAGGCCATCAAGAA-3′, Reverse 5′-GCTCCCCTCTGGTTTTAAGG-3′ [22]; GAPDH: Forward 5′-GTCATCCATGACAACTTTGG-3′, Reverse 5′-GAGCTTGACAAAGTGGTCGT-3′ [23]. The PCR protocol consisted of thermal cycling as follows: initial denaturation at 95°C for 2 min, followed by 40 cycles of 95°C for 20 s, 58°C for 30 s, and 72°C for 30 s using an Eppendorf epGradient Mastercycler (Eppendorf, Hamburg, Germany). In all experiments, two negative controls were carried through all steps. Data quantitation was performed using the relative standard curve method [24].

### Western Blotting

Protein extracts were separated on SDS-PAGE and electroblotted onto PVDF membranes according to standard procedures. The membranes were blocked with 5% nonfat dry milk and incubated with the respective primary antibodies: AKT, p-AKT, c-Myc, ERK, JNK, MEK, p38, and GAPDH obtained from Santa Cruz Biotechnology Inc. (Santa Cruz, CA) as well as p21, p27, p-ERK, p-JNK, p-MEK, p-p38, p-STAT1(Y701), p-STAT1(S727), STAT1, p-STAT3(Y705), p-STAT3(S727), STAT3, p-STAT5(Y694) and STAT5 purchased from Cell Signaling Technology (Danvers, MA). Western blotting were visualized with HRP-conjugated secondary antibodies (Jackson ImmunoResearch Laboratories, Inc., West Grove, PA) followed by enhanced chemiluminescence detection (Biological Industries, Beit Haemek, Israel).

### Virus Production and Transduction of Cells

A human pLKO.1 lentiviral STAT1 shRNA set (RHS4533, including 5 clones) and pLKO.1 empty lentiviral vector (RHS4080) were purchased from Open Biosystems. The pLKO.1 Non-target shRNA control plasmid was purchased from Sigma. Infectious Lentivirus was produced by cotransfecting 293T cells with shRNA-expressing lentiviral plasmids together with the packaging plasmid pRΔ8.9 and envelope plasmid pMD.G. Supernatants containing infectious virus particles were collected 48 h after transfection and then used to transduce HL60 cells by spinoculation in the presence of polybrene (6 µg/mL). Positive transductants were selected with puromycin, and then the expression of STAT1 was analyzed by Western blotting.

### Statistical Analysis

The statistical significance of differences between groups was evaluated by the unpaired Student’s t test and indicated with *** P<.001, **/## P<.01, */# P<.05. All statistical tests were two-sided.

## Results

### Dasatinib Induces Differentiation of AML Cells

**Figure 1:**
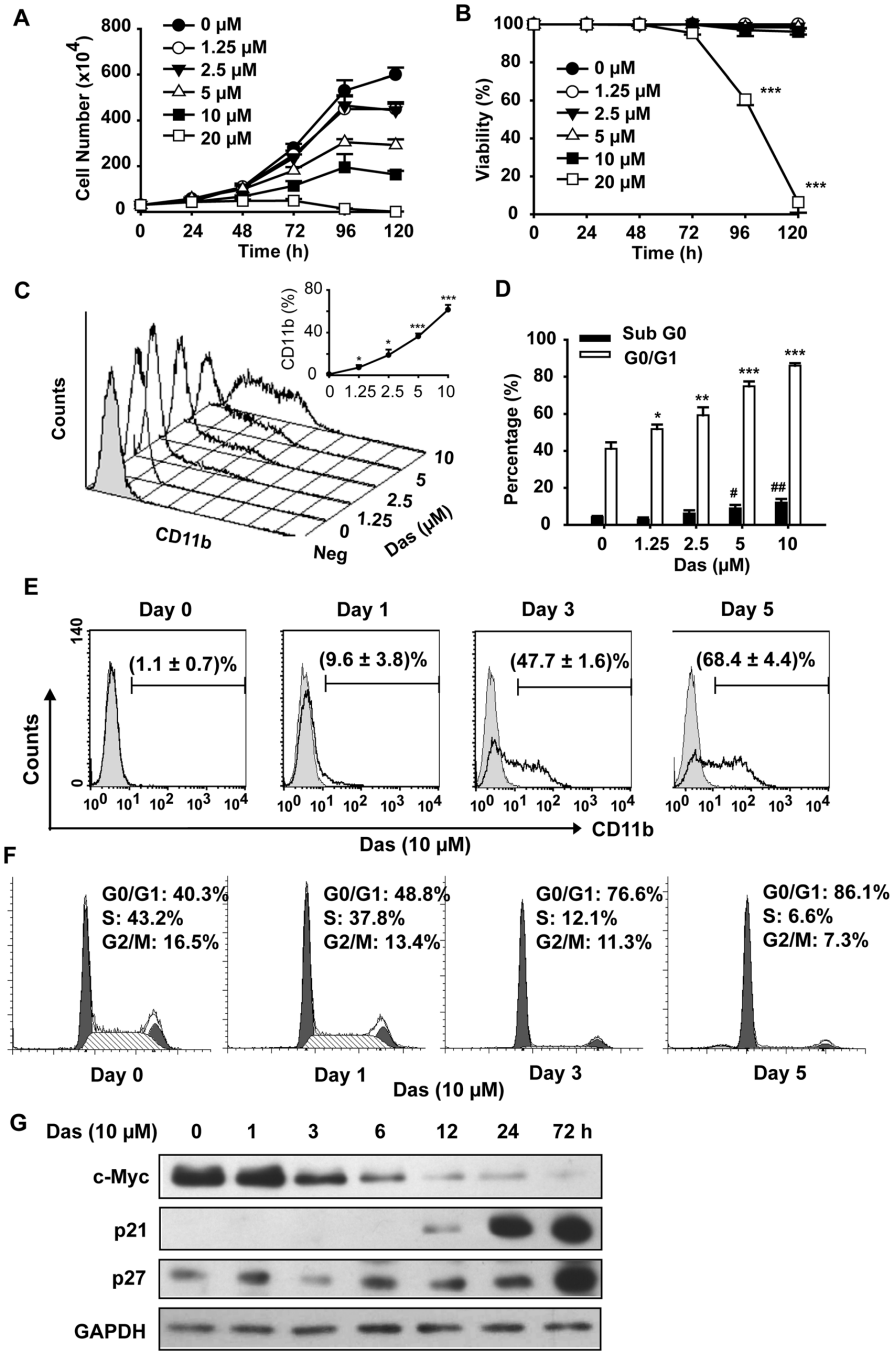
Dasatinib exhibits dose- and time-dependent effect on inducing differentiation of HL60 cells. HL60 cells were treated with dasatinib for the indicated times. Aliquots of the cultures were withdrawn at each time point and subjected to manual counting, following trypan blue staining for determination of (A) the total number of viable cells and (B) the percentage of viable cells. Data presented are the mean ± SD of cell numbers from triplicate wells. The experiments were repeated twice. *** P<.001 versus vehicle treated cells. (C) and (E) The percentages of CD11b expression cells were analyzed as described. Data presented are the mean ± SD of three independent experiments. *** P<.001 and * P<.05 versus vehicle treated cells. (D) and (F) Cell cycle proportion was analyzed by flow cytometer, following propidium iodide staining. Data presented are the mean ± SD of three independent experiments. *** P<.001, **/## P<.01, */# P<.05 versus vehicle treated cells. (G) Cell extracts were subjected to Western blotting analysis with specific antibodies. The results are representative of three independent experiments.

Exponentially growing HL60 cells were treated with different concentrations of dasatinib (1.25–20 µM) with indicated exposure times. Compared with control group, dasatinib significantly inhibited the proliferation of HL60 cells in a dose- and time-dependent manner (Fig. 1A). Interestingly, we found that dasatinib exhibited little effect on viability of HL60 cells except high concentration (20 µM), at which the apoptosis of HL60 cells was observed (Fig. 1B and data not shown). To explore the anti-neoplastic effect of dasatinib within the concentration of 1.25–10 µM, we studied its effect on triggering cell differentiation and cell cycle arrest after 120 h treatment (Fig. 1C and D). As shown in Fig. 1C, dasatinib induced the expression of CD11b, a myeloid differentiation marker, in a dose-dependent manner. In consistence with previous study [17], dasatinib-induced differentiation was paralleled by G0/G1 phase arrest (Fig. 1D).

To study the progression of dasatinib-induced differentiation, HL60 cells were treated with dasatinib (10 µM) for 0–120 h. Of note, dasatinib-induced CD11b expression was already detectable as early as 24 h, albeit slightly, with approximately 10% positive cells. After that the population increased to 48% and 68% at 72 h and 120 h, respectively (Fig. 1E),and even reached >90% at day 11 (data not shown). Moreover, dasatinib-induced G0/G1 phase arrest was also in line with the differentiation progression, which was detectable at 24 h of treatment (Fig. 1F). Consistently, dasatinib significantly decreased the expression of c-Myc which is involved in leukemic cell proliferation and increased the expression of p21 and p27 which play important roles in preventing cell cycle progression (Fig. 1G).

**Figure 2:**
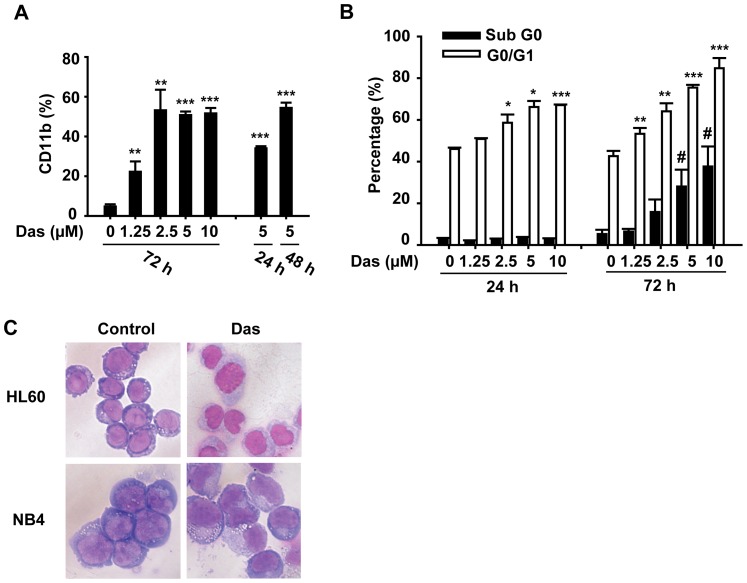
Dasatinib induces differentiation of NB4 cells. NB4 cells were treated with different concentrations of dasatinib for the indicated times. (A) The percentages of CD11b expression cells were determined as described. Data presented are the mean ± SD of three independent experiments. *** P<.001, ** P<.01 versus vehicle treated cells. (B) Cell cycle proportion was analyzed as described. Data presented are the mean ± SD of three independent experiments. *** P<.001, ** P<.01, */# P<.05 versus vehicle treated cells. (C) HL60 cells were treated with dasatinib (10 µM) for 72 h and NB4 cells were treated with dasatinib (5 µM) for 48 h. Cells were collected and cytospined onto slides, then stained with Wright–Giemsa stain. Stained cells were observed under microscope (×40). The data are representative of three independent experiments.

The differentiation-inducing capacity of dasatinib was further determined on another AML cell line NB4 cells. Dasatinib-induced expression of CD11b was in a dose-dependent manner at low concentrations (1.25 and 2.5 µM). However, high concentrations (5 and 10 µM) of dasatinib did not induce an enhanced expression of CD11b (Fig. 2A). This could be attributed to dasatinib-induced increase of hypodiploid cells at 72 h. Upon high concentrations (5 and 10 µM) of dasatinib treatment, approximately more than 30% of NB4 cells were accumulated in Sub G0 phase. Actually, dasatinib-induced cell-cycle blockade was manifested before the accumulation of Sub G0 phase cells. An obvious G0/G1 phase arrest was already detectable at 24 h after dasatinib treatment (2.5–10 µM), when no hypodiploid cells were observed (Fig. 2B). Furthermore, the ability of dasatinib to induce AML differentiation was also confirmed by morphologic changes of HL60 and NB4 cells, namely a reduction of nucleus to cytoplasm ratio and an obvious nuclear segmentation compared with control group (Fig. 2C). Taken together, these results indicated that dasatinib exerted a differentiation effect on AML cells.

### Dasatinib Induces the Activation of STAT1

**Figure 3:**
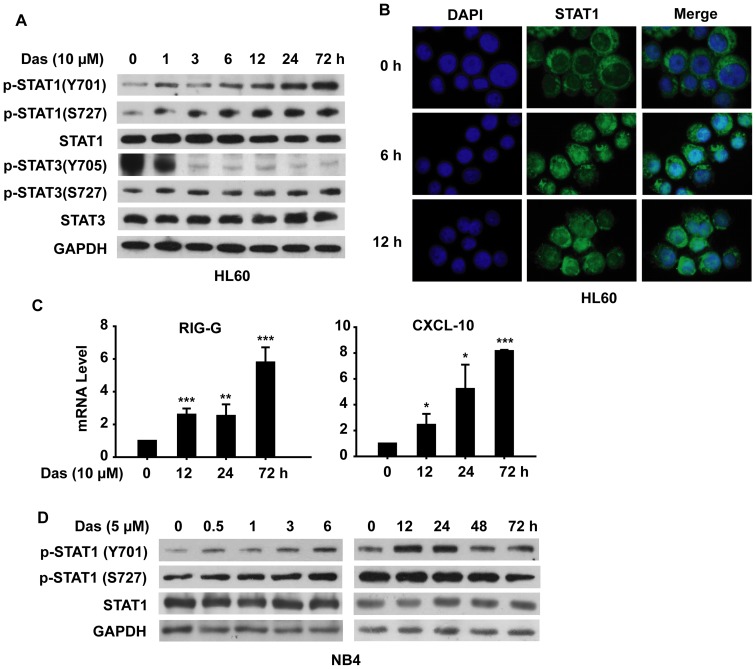
Dasatinib induces activation of STAT1. HL60 cells were treated with dasatinib (10 µM) for the indicated times. (A) Whole cell lysates were analyzed by SDS-PAGE followed by Western blotting using specific antibodies. The experiments were repeated three times and the data show the representative results. (B) Representative fluorescence microphotographs showed a translocation of STAT1 from cytoplasm to nucleus. Original magnification, ×63. The results are representative of three independent experiments. (C) The expression of CXCL-10 and RIG-G and IRF-1 mRNA was determined by real-time quantitative PCR. The expression of GAPDH was used as the internal control gene. Shown are fold increases over untreated cells. Data presented are the mean ± SD of three independent experiments. *** P<.001, ** P<.01, * P<.05 versus untreated cells. (D) NB4 cells were treated with dasatinib (5 µM) for the indicated times. The level of p-STAT1 (Y701), p-STAT1 (S727), STAT1 and GAPDH proteins was determined by Western blotting analysis. The results are representative of three independent experiments.

Numerous reports have demonstrated the essential role of STAT proteins in myeloid differentiation; here we intended to determine whether dasatinib-induced AML differentiation was mediated by STAT signal pathway. HL60 cells were incubated with dasatinib (10 µM) for indicated times and then analyzed for the phosphorylation of STAT proteins. As shown in Fig. 3A, dasatinib treatment led to an increased phosphorylation of STAT1 (Y701 and S727) within 1 h, which was then augmented with time, whereas dasatinib significantly decreased the phosphorylation of STAT3 (Y705), which was almost undetectable after 3 h treatment. In addition, dasatinb exhibited little effects on the phosphorylation of STAT3 (S727) and STAT5 (Y694) (Fig. 3A and data not shown). Given that phosphorylated STAT1 will translocate into the nucleus to exert its gene regulation effects, we then determined the localization of STAT1 by immunofluorescence assay. In the absence of dasatinib, STAT1 was predominantly cytoplasmic, while dasatinib treatment induced the redistribution of STAT1 from the cytoplasm to the nucleus (Fig. 3B). To further confirm the ability of phosphorylated STAT1 on gene transcription regulation, the expressions of two direct STAT1 target genes (RIG-G and CXCL-10) [25], [26] were tested by real time PCR. As shown in Fig. 3C, the transcription of both genes was enhanced by dasatinib in a time-dependent manner. In consistence with these results, dasatinib-induced activation of STAT1 was also observed in NB4 cells. A modest increase of p-STAT1 (Y701) and p-STAT1 (S727) were virtually detectable at 0.5 h after dasatinib treatment, but the level of p-STAT1 (Y701) returned to baseline by 48 h, while the level of p-STAT1 (S727) returned to baseline by 12 h (Fig. 3D). These results indicated that STAT1 might play a role in dasatinib-induced differentiation of AML cells.

### STAT1 knockdown Inhibits Dasatinib-induced Differentiation

**Figure 4:**
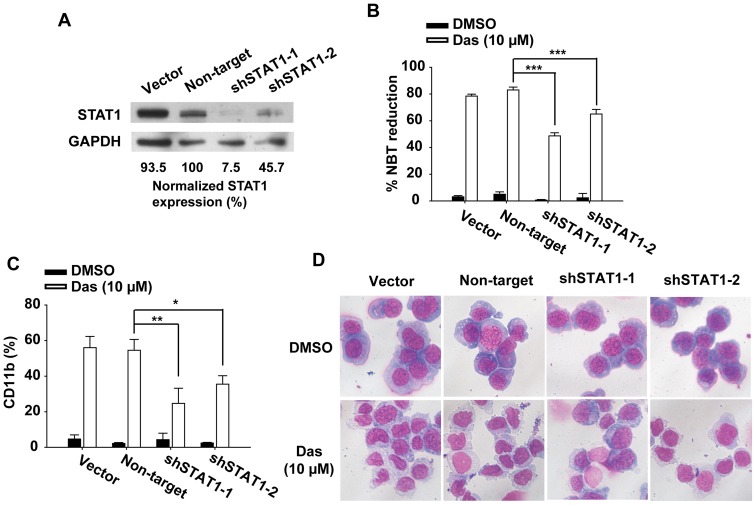
Silencing of STAT1 expression by shRNA inhibits dasatinib-induced differentiation of HL60 cells. HL60 cells transduced with empty vector, Non-target shRNA or STAT1 shRNA (shSTAT1-1 and shSTAT1-2) were treated with dasatinib (10 µM) for 72 h. (A) STAT1 expression was detected by Western blotting. Band intensities were quantified using Quantity One 4.4.0 software (Bio-Rad) and then normalized to the amount of GAPDH in each sample. All samples were compared with the signal detected in Non-target shRNA transduced cells. The data are representative of three independent experiments. (B) NBT reduction assay was performed as described. The percentage of NBT-positive cells was calculated by counting at least 200 cells under a light microscope. Data presented are the mean ± SD of three independent experiments. *** P<.001 (C) CD11b expression was measured by flow cytometer. Data presented are the mean ± SD of three independent experiments. ** P<.01, * P<.05 (D) Cells were collected onto slides by cytospin, stained by Wright–Giemsa stain and observed under microscope. Original magnification, ×40. The data are representative of three independent experiments.

The finding that STAT1 was significantly activated in AML cells after dasatinib treatment prompted us to investigate whether STAT1 contributed to dasatinib-induced differentiation. The endogenous expression of STAT1 was knocked down by shRNA. Of the 5 shRNA clones, two shRNA sequences that exhibited the strongest knockdown efficiency were selected to silence STAT1 expression in HL60 cells. As shown in Fig. 4A, expression of shSTAT1-1 and shSTAT1-2 significantly reduced STAT1 expression in HL60 cells compared with Vector or Non-target shRNA. As expected, silencing of STAT1 expression significantly inhibited dasatinib-induced AML differentiation. In detail, STAT1-knockdown HL60 cells showed markedly reduced NBT positive cells after dasatinib treatment compared with Vector and Non-target shRNA-expressing cells (78.5±1.4, 83.1±2.1, 48.7±2.3, and 65.0±3.4 for Vector, Non-target shRNA, shSTAT1-1, and shSTAT1-2, respectively; Fig. 4B). STAT1-knockdown HL60 cells also demonstrated significantly reduced CD11b expression after dasatinib treatment compared with Vector and Non-target shRNA-expressing cells (56.0±6.3, 54.5±6.1, 24.7±8.5, and 36.5±4.7 for Vector, Non-target shRNA, shSTAT1-1, and shSTAT1-2, respectively; Fig. 4C). Moreover, STAT1 shRNA-transduced cells exhibited less mature granulocyte morphology compared with Vector and Non-target shRNA-transduced cells, as evidenced by a decreased ratio of cytoplasm to nuclear (Fig. 4D). These results indicated that knockdown of STAT1 in HL60 cells resulted in a significantly decreased differentiation response to dasatinib stimulation.

### Dasatinib Induces Activation of MEK/ERK Cascade

**Figure 5:**
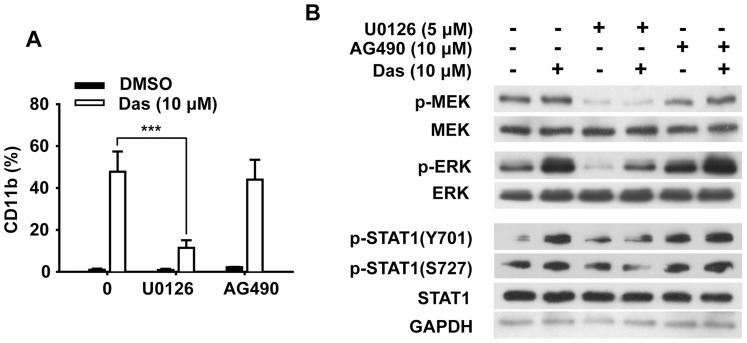
Dasatinib-induced HL60 cells differentiation could not be abrogated by JAK inhibitor AG490. HL60 cells were treated with dasatinib (10 µM) in the presence of U0126 (5 µM) or AG490 (10 µM) for 72 h. (A) The percentages of CD11b expression cells were determined as described. Data presented are the mean ± SD of three independent experiments. *** P<.001 (B) The expression of p-MEK, p-ERK, p-STAT1(Y701) and p-STAT1(S727) as well as their total proteins were analyzed by Western blotting. The experiments were repeated three times and the data show the representative results.

**Figure 6:**
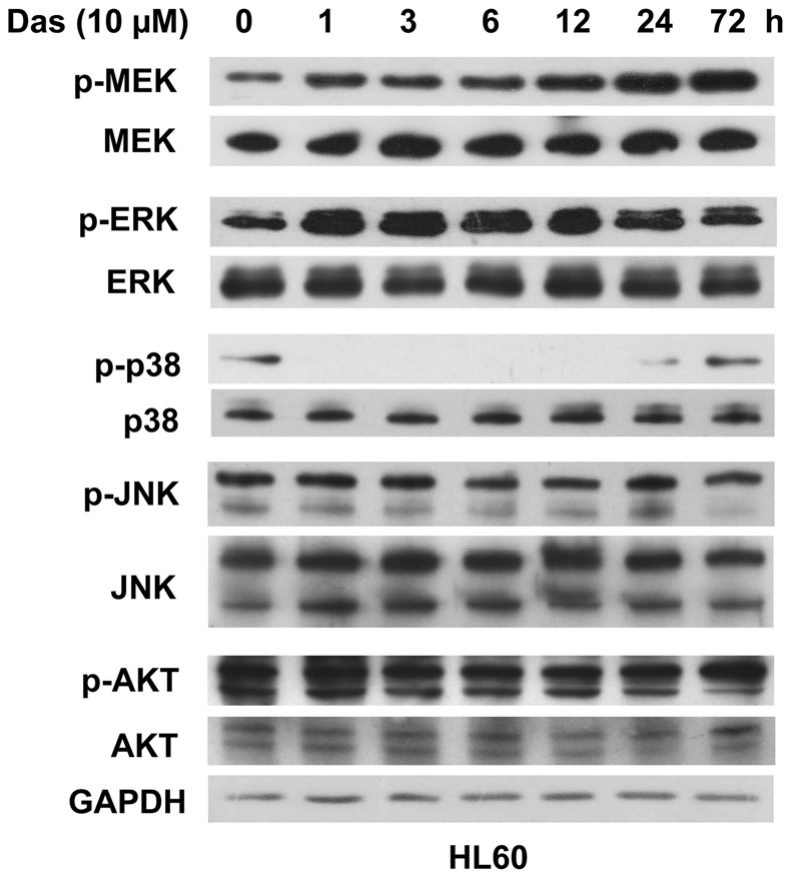
Dasatinib enhances the phosphorylation of MEK/ERK cascades. HL60 cells were treated with dasatinib (10 µM) for the indicated times. The expressions of phosphorylated MEK, ERK, p38, JNK and AKT as well as their total proteins were analyzed by Western blotting. All the experiments were repeated three times and the data show the representative results.

Given that STAT1 is phosphorylated at tyrosine 701 by the Janus kinase (JAK) family members and at serine 727 by MAPK cascades including MEK, ERKs, p38 and JNKs [27], [28], [29] as well as PI3K-AKT pathway [30], we first evaluated the effects of dasatinib on JAK1, JAK2 and Tyk2 kinases. Unexpectedly, dasatinib had no effect on the phosphorylation of JAK1 or JAK2 (data not shown). In addition, neither dasatinib-induced CD11b expression nor dasatinib-enhanced phosphorylation (Y701 and S727) of STAT1 was affected by the JAK inhibitor AG490 (Fig. 5). In contrast, the phosphorylation of MEK was augmented within 1 h and increased to a high level at 72 h of dasatinib treatment (Fig. 6). In consistence, the enhanced phosphorylation of ERK was also detectable as early as 1 h and then maintained at a high level for at least 72 h. We then determined the effect of dasatinib on the phosphorylation of other MAPK family members, p38 and JNK. As shown in Fig. 6, phosphorylation of p38 was transiently abolished within 1 h, and then returned to baseline at 72 h of dasatinib treatment, while dasatinib exhibited little effects on the phosphorylation of JNK and AKT.

### Activation of STAT1 is Dependent on MEK/ERK Activation

**Figure 7:**
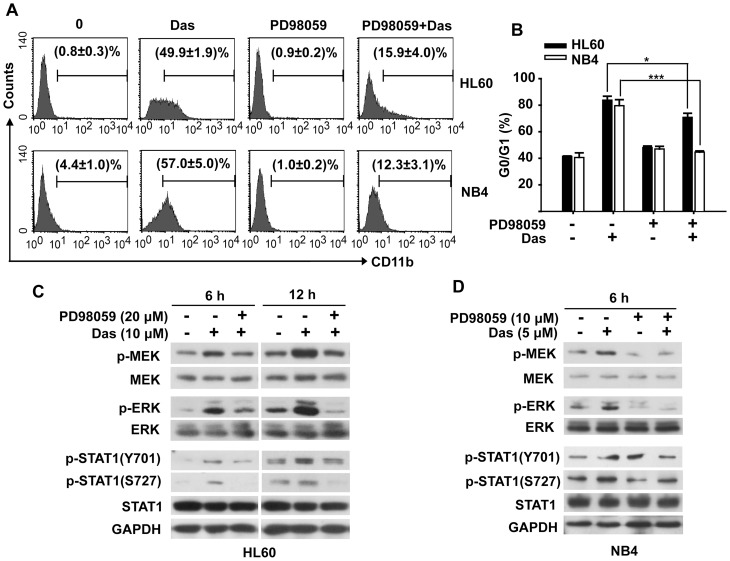
Dasatinib-induced AML differentiation requires MEK/ERK-dependent activation of STAT1. HL60 cells were treated with dasatinib (10 µM) in the presence of PD98059 (20 µM) for 72 h, and NB4 cells were treated with dasatinib (5 µM) in the presence of PD98059 (10 µM) for 48 h. HL60 and NB4 cells were pretreated with PD98059 for 1 h, and then incubated with dasatinb. (A) The percentages of CD11b expression cells were determined as described. Data presented are the mean ± SD of three independent experiments. (B) Cell cycle proportion was analyzed as described. Data presented are the mean ± SD of three independent experiments. *** P<.001, * P<.05. (C) and (D) The expression of p-MEK, p-ERK, p-STAT1(Y701) and p-STAT1(S727) as well as their total proteins in HL60 and NB4 cells were analyzed by Western blotting. The experiments were repeated three times and the data show the representative results.

To determine whether the phosphorylation of MEK/ERK cascade was indispensible for dasatinib-induced AML differentiation, HL60 and NB4 cells were treated with dasatinib in the presence of MEK inhibitor PD98059 and then analyzed for CD11b expression. The concentration of PD98059 to treat HL60 and NB4 were 20 and 10 µM, respectively, at which no cytotoxicity was observed in both cell lines when treated with PD98059 alone or in combination with dasatinib (data not shown). As shown in Fig. 7A, PD98059 significantly inhibited dasatinib-induced CD11b expression in both cells. In detail, the population of CD11b-positive cells decreased from 50% to 16% in HL60 cells and from 57% to 12% in NB4 cells in the presence of PD98059, respectively. In addition, PD98059 also significantly inhibited dasatinib-induced G0/G1 phase arrest, especially in NB4 cells of which the percentage of G0/G1 phase cells decreased from 80% to 40% (Fig. 7B). As expected, the activation of MEK and ERK was successfully inhibited by PD98059 (Fig. 7C and D). Interestingly, the phosphorylation of STAT1 on both sites (Y701 and S727) was strikingly inhibited by PD98059 in HL60 and NB4 cells. These results were further corroborated by another MEK inhibitor U0126. Dasatinib-induced CD11b expression decreased from 50% to 11% in the presence of U0126 (Fig. 5A). Western blotting analysis also confirmed that U0126 not only inhibited dasatinib-induced serine phosphorylation of STAT1 (S727), but also abrogated the tyrosine phosphorylation of STAT1 (Y701) (Fig. 5B). Taken together, dasatinib-induced activation of STAT1 was dependent on MEK/ERK activation, thus leading to the differentiation of leukemic cells.

## Discussion

The present study demonstrated the capacity of dasatinib to induce leukemic cell differentiation, as evidenced by the enhanced expression of CD11b, G0/G1 phase arrest and the decreased ratio of nuclear to cytoplasm in dasatinib-treated AML cells. Notably, we found that the biological function of dasatinib on inducing AML differentiation was mediated by MEK/ERK dependent activation of STAT1.

STAT family proteins, activated by cytokines, hormones, and growth factors function as transcription factors that mediate a variety of biologic processes, such as cell proliferation and apoptosis [31]. Among at least seven members, STAT1 has been reported to play essential roles in myeloid differentiation. STAT1 activation was first identified in ATRA-induced myeloid differentiation of various leukemia cell lines [32]. In addition, STAT1 was also reported to be activated by Bryostatin 1 to mediate the differentiation of chronic lymhocytic leukemia cells (CLL). Here we observed a significant increase of tyrosine and serine phosphorylation of STAT1 after dasatinib treatment, accompanied by the translocation of STAT1 from the cytoplasm to the nucleus, thus leading to the enhanced transcription of STAT1 target genes. Silencing of STAT1 expression by shRNA significantly lessened dasatinib-induced differentiation, suggesting an essential role of STAT1 in dasatinib’s differentiation effect. In addition, the phenomenon that the phosphorylation of STAT1 (Y701 and S727) returned to basal level or even lower in NB4 cells at late time after dasatinib treatment might be resulted from intracellular signals that triggered apoptosis of NB4 cells. In contrast to the tumor suppressor function of activated STAT1, STAT3 is mainly identified as an oncogene [33], [34]. Constitutive tyrosine and serine phosphorylation of STAT3 has been demonstrated in many different malignancies, including AML [35], [36]. Inhibition of STAT3 activation induces apoptosis in AML cell lines and primary samples [36]. However, the important roles for STAT3 in granulocytic differentiation have also been reported in vitro and in vivo studies [37], [38]. Thus, the exact role of STAT3 in leukemogenesis remains controversial. In our study, the tyrosine phosphorylation of STAT3 was significantly down-regulated by dasatinib, while the serine phosphorylation of STAT3 was unaffected. A previous study has demonstrated that constitutive phosphorylation of STAT3 on serine 727, not tyrosine 705, was required for DNA-binding and transcription activation of target genes for survival [39]. Thus the serine phosphorylation of STAT3 here might disrupt apoptotic signal pathway caused by the decrease of tyrosine phosphorylation, thereby conferring a survival advantage that was necessary for differentiation of HL60 cells.

JAK1, JAK2, and Tyk2, which are reported to directly phosphorylate STAT1 protein at tyrosine site (Y701) following activation of the IFN receptors by their respective ligands [31], [40], did not seem to be potential targets for dasatinib. In detail, dasatinib did not cause any changes in the levels of JAK1, JAK2 or Tyk2 phosphorylation and neither dasatinib-induced CD11b expression nor dasatinib-enhanced phosphorylation of STAT1 (Y701 or S727) was affected by JAK inhibitor AG490. Previous studies have shown that STAT1 was phosphorylated at serine site by several kinases, including MEK, ERK, JNK, p38 kinases and PI3K/AKT pathway. Here we found that MEK and ERK were significantly activated in dasatinib treated AML cells. However, the phosphorylation of p38 was totally abrogated by dasatinib except a recovery at 72 h and the phosphorylation of JNK and AKT were hardly changed after dasatinib treatment. We extended those observations to show that activation of MEK/ERK cascade was indispensible for dasatinib’s differentiating effect. Critically, we showed that dasatinib-induced expression of CD11b and G0/G1 arrest were significantly inhibited by MEK inhibitors PD98059 and U0126. In line with these facts, dasatinib-induced phosphorylation of STAT1 (both tyrosine 701 and serine 727 sites) were inhibited by MEK inhibitors, indicating MEK/ERK-dependent STAT1 activation might be indispensable for the differentiation effect of dasatinib.

SFKs have been reported to activate the Ras/Raf/MEK/ERK pathway via recruitment of Shc and Shp2 proteins [41]. Here we observed that dasatinib, as an efficient SFKs inhibitor, did not inhibit the MEK/ERK cascades in AML cells. On the contrary, dasatinib significantly induced the phosphorylation of MEK and ERK, which was approved to be indispensible for dasatinib’s differentiating effect. This phenomenon could be partially explained by the fact that dasatinib is also a weak RAF inhibitor. Packer et al. [42] reported that the binding of weak RAF inhibitors (imatinib, nilotinib and dasatinib) to BRAF and CRAF drives BRAF:CRAF heterodimer as well as BRCF and CRAF homodimer formation in the presence of oncogenetic RAS, thus leading to paradoxical activation of both BRAF and CRAF, followed by the activation of MEK and ERK. Considering the complex mechanisms involved in activation of Ras-Raf-MEK-ERK pathway, further studies will be performed to better understand the effect of dasatinib-induced activation of MEK/ERK kinases in our future work.

In summary, the present results demonstrated for the first time that the differentiation-induction effect of dasatinib required the activation of STAT1, which depended on MEK/ERK cascades activation. Our results suggest that attempts to treat AML with a combination of dasatinib and MEK inhibitors should be made with great caution. The identification of potential molecular mechanisms for dasatinib’s biological effect on AML differentiation may provide some molecular theoretical basis for clinical application when dasatinib is combined with other target based drugs in leukemia therapy.
